# Application of Statistically Based Experimental Designs to Optimize Cellulase Production and Identification of Gene

**DOI:** 10.1007/s13659-014-0046-y

**Published:** 2014-11-23

**Authors:** Aarti Thakkar, Meenu Saraf

**Affiliations:** Department of Microbiology, University School of Science, Gujarat University, Ahmedabad, 380 009 Gujarat India

**Keywords:** *Bacillus amyloliquefaciens*, Cellulase gene, Optimization, Plackett–Burman design, Response surface methodology

## Abstract

**Abstract:**

A natural bacterial strain identified as *Bacillus amyloliquefaciens* MBAA3 using 16S rDNA partial genome sequencing has been studied for optimization of cellulase production. Statistical screening of media components for production of cellulase by *B. amyloliquefaciens* MBAA3 was carried out by Plackett–Burman design. Plackett–Burman design showed CMC, MgSO_4_ and pH as significant components influencing the cellulase production from the media components screened by Plackett-Burman fractional factorial design. The optimum concentrations of these significant parameters were determined employing the response surface central composite design, involving three factors and five levels was adopted to acquire the best medium for the production of cellulase enzyme revealed concentration of CMC (1.84 g), MgSO_4_ (0.275 g), and pH (8.5) in media for highest enzyme production. Response surface counter plots revealed that middle level of MgSO_4_ and middle level of CMC, higher level of CMC and lower level of pH and higher level of MgSO_4_ with lower level of pH increase the production of cellulase. After optimization cellulase activity increased by 6.81 fold. Presence of cellulase gene in MBAA3 was conformed by the amplification of genomic DNA of MBAA3. A PCR product of cellulase gene of 1500 bp was successfully amplified. The amplified gene was conformed by sequencing the amplified product and sequence was deposited in the gene bank under the accession number KF929416.

**Graphical Abstract:**

Response surface graph showing interaction effects between concentration of **a** CMC and MgSO4. **b** pH and CMC. **c** MgSO4 and pH
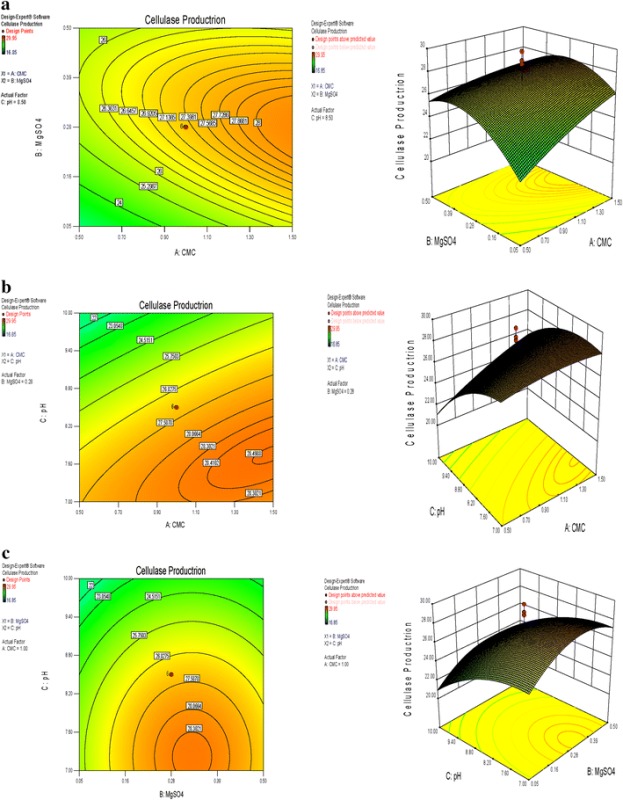

## Introduction

Cellulases (1,4-(1,3;1,4)-β-D-glucan 4-glucanohydrolase) refers to a group of enzymes which hydrolyze cellulose. Cellulose is the most abundant organic source of food, fuel and chemicals [1]. It is the primary product of photosynthesis in terrestrial environments and the most abundant renewable bioresource produced in the biosphere [2]. Cellulase is an enzyme system that consists of at least three different components, i.e. (i) endoglucanase or 1,4-β-d-glucan-4-glucanohydrolases (EC 3.2.1.4) (ii) exoglucanases including 1,4-β-d-glucan glucanohydrolases (cellodextrinases; EC 3.2.1.74) and 1,4-β-d-glucan cellobiohydrolases (cellobiohydrolases; EC 3.2.1.91), and (iii) β-glucosidases or β-glucoside glucohydrolases (EC 3.2.1.21) [3]. Although cellulases are distributed throughout the biosphere, they are mostly found in microbial sources. Cellulases are inducible enzymes synthesized by a large diversity of microorganisms including both fungi and bacteria during their growth on cellulosic materials [1]. Bacteria which have high growth rate as compared to fungi have good potential to be used in cellulase production. The cellulolytic property of some bacterial genera such as *Cellulomonas, Cellvibrio, Pseudomonas* sp, *Bacillus,* *and Micrococcus* has been also reported. Enzyme production is closely controlled in microorganisms and for improving its productivity these controls can be ameliorated. Cellulase yields appear to depend upon a complex relationship involving a variety of factors like inoculums size, pH value, temperature, presence of inducers, medium additives, aeration, growth time, and so forth [4].

Microbial cellulases have potential application in various industries including pulp and paper, textile, laundry, biofuel production, food and feed industry, brewing and agriculture, they are used in animal feeds for improving the nutritional quality and digestibility. A potential challenging area where cellulases would have a central role is the bioconversion of renewable cellulosic biomass to commodity chemicals [5]. Productivity of microbial enzymes and metabolites can be increased by manipulating nutritional requirements, physical parameters and genetic make up of the producing strain. Production cost is considered the bottleneck of many biotechnological processes [6]. Development of economical medium requires selection of carbon, nitrogen, phosphorous, potassium and trace element sources. Nutritional requirement can be manipulated by the conventional or statistical methods. Conventional method involves changing one independent variable at a time while keeping the others at fixed level. However, statistical method offers several advantages over conventional method being rapid and reliable, short lists significant nutrients, helps understanding the interactions among the nutrients at various concentrations and reduces the total number of experiments tremendously resulting in saving time, glassware, chemicals and manpower [7]. Initial screening of the ingredients is done to understand the significance of their effect on the product formation and then a few better ingredients are selected for further optimization [8]. Response surface methodology (RSM) is a useful tool which integrates mathematical and statistical approaches to analyze the effects of defined independent variables on the response without the need for prior knowledge of a predetermined relationship between the response function and the variables [9]. RSM is now considered as a standard statistical approach designing experiments, building models, evaluating the effects of many factors and finding the optimal conditions for desirable responses and reducing the number of required experiments [10]. In the present study, media components such as CMC, MgSO_4_ peptone, pH etc. were evaluated by Plackett–Burman design and further optimized using RSM for increasing cellulase production from our isolate *B. amyloliquefaciens* MBAA3. The role of each variable, their interactions and statistical analysis for cellulase production were explained by applying the second-order polynomial model. The analysis was done using software Design-Expert version 8.0. Further, this study describes the identification of gene encoding for this enzyme and the determination of its nucleotide sequence.

## Results and Discussions

### Isolation and Identification

**Fig. 1:**
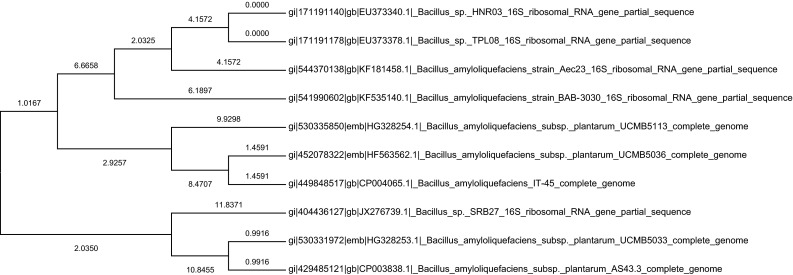
Phylogenetic relationship on the basis of homology index for bacterial isolate *B. amyloliquefaciens* MBAA3

Bacterial culture isolated from farm soil samples collected from Ahmedabad, Gujarat, India on N. agar (Himedia, Mumbai, India) was screened for cellulase production on CMC agar plate. Genomic DNA of the isolate was extracted and the 16S rDNA gene was amplified using universal primers. The purified PCR product was sequenced and the phylogenic relationship of the isolate was determine by comparing the sequence data with the existing sequences available through the gene bank database of the National Center for Biotechnology Information (NCBI, Bethesda, MD, USA) and identified as *Bacillus amyloliquefaciens* denoted as MBAA3 and the sequence was submitted in the gene bank under the accession number KF535140. The phylogenetic tree as shown in Fig. 1 was drawn using bioinformatics software MEGA 4.0.

### Statistical Optimization of Cellulase Production from MBAA3

The chemical composition of the culture medium and environmental factors influence cell growth and cellulase production [6]. A better understanding of the medium components and environmental factors and their optimal control can, therefore, be used to improve the cellulase enzyme production. A statistical method was applied for studying different factors affecting cellulase production by *B. amyloliquefaciens* isolate MBAA3. Plackett-Burman design was used to screen eight different medium components as 12 run experiment with two level of concentration of each variable The independent variables and their respective high and low concentrations used in the optimization study are represented in Table 1, whereas the Plackett-Burman experimental design for 12 trials with two level of concentration of each variable is given in Table 2, which was followed for the optimization of medium components for cellulase production. The variables A–H represented the medium constituents and I–K represented the dummy variables/unassigned variables. The results of Plackett-Burman experiment with respect to cellulase production, the effect, standard error, t (xi), p, and confidence level of each component are represented in Table 5. The components were screened at a confidence level of 95 % on the basis of their effects. When components show significance at or above 95 % confidence level and it’s effect is negative, it is considered effective for production but the amount required may be lower than the indicated as low (−1) concentration in Plackett-Burman experiment. If the effect is found positive, a higher concentration than the indicated high value (+) concentration is required. In our experiment CMC, MgSO_4_ and pH gave confidence level > 95 % and could be considered significant. Remaining components as sucrose, yeast extract, peptone, K_2_HPO_4_, and temperature showed confidence level < 95 % and were considered insignificant in the study. The t-value and probability value (*p* value) is a tool for evaluating the significance and contribution of each of the parameters to the statistical polynomial model equation. The pattern of interactions between the variables is indicated by these coefficients. The larger the magnitude of t test and the smaller the p-value are an indication of high significance of the corresponding coefficient [11]. Variables with very low probability levels (close to 0.00) contribute to the model, while others can be neglected and eliminated from the model. The p-value suggested that the coefficient for the linear effect of CMC, MgSO_4_ and pH were most significant.

**Table 1:** Medium components and their variables used in Placket-Burman design for cellulase production using *Bacillus amyloliquefaciens* strain MBAA3

Variable	Medium component	+Value	−Value
A	CMC	1.0	0.1
B	Sucrose	0.2	0.02
C	Yeast extract	0.2	0.02
D	peptone	0.5	0.05
E	K_2_HPO_4_	0.1	0.01
F	MgSO_4_	0.1	0.01
G	Temp.	37	28
H	pH	5	9

**Table 2:** Plackett-Bueman design generated by fractional rotation of full factorial design where A to H is independent variable and I to K is dummy variables

Run	A	B	C	D	E	F	G	H	I	J	K	U/mL
1	+	+	−	+	+	+	−	−	−	+	−	30.54
2	−	+	+	−	+	+	+	−	−	−	+	1.53
3	+	−	+	+	−	+	+	+	−	−	−	46.65
4	−	+	−	+	+	−	+	+	+	−	−	4.02
5	−	−	+	−	+	+	−	+	+	+	−	37.41
6	−	−	−	+	−	+	+	−	+	+	+	5.23
7	+	−	−	−	+	−	+	+	−	+	+	25.21
8	+	+	−	−	−	+	−	+	+	−	+	45.62
9	+	+	+	−	−	−	+	−	+	+	−	1.23
10	−	+	+	+	−	−	−	+	−	+	+	1.65
11	+	−	+	+	+	−	−	−	+	−	+	14.78
12	−	−	−	−	−	−	−	−	−	−	−	9.25

**Table 3:** Experimental range and levels of the independent variables of selected components used for response surface central composite design

Variable	Components	−α	−1	0	+1	+α
A	CMC	0.159104	0.5	1.0	1.5	1.840896
B	MgSO_4_	−0.1034	0.05	0.275	0.5	0.653403
C	pH	5.977311	7	8.5	10	11.02269

**Table 4:** Steps and conditions of thermal cycling for PCR

Steps	Temperature	Time	Cycles
Initial denaturation	95 °C	2 min	1
Final denaturation	94 °C	30 Sec	30
Annealing	58 °C	30 Sec	
Extention	72 °C	90 Sec	
Final extention	72 °C	10 min	1

**Table 5:** Statistical analysis of components by Plackett-Burman design for cellulase production by *Bacillus amyloliquifaciens* strain MBAA3

Component	Effect	Standard error	T-value	*P* Value	Confidence (%)
CMC	17.49	3.963858	4.412368	0.0216	97.83997
Sucrose	8.99	5.04375	1.782404	0.1727	82.72997
Yeast extract	2.778333	5.04375	0.550847	0.620106	37.98936
peptone	2.896667	5.04375	0.574308	0.605995	39.40054
K2HPO_4_	0.643333	5.04375	0.127551	0.906574	9.342577
MgSO_4_	18.47333	5.04375	3.662619	0.035181	96.48193
Temperature	9.23	5.04375	1.829988	0.164672	83.53282
pH	16.33333	5.04375	3.238331	0.047912	95.20884

The central composite design (CCD) was employed to study the interaction among the significant factors and also determine their optimal levels. In the present work, experiments were planned to obtain a quadratic model consisting of 20 trials. The plan includes 20 experiments and two levels of concentration for each factor. In order to study the combined effect of these variables, experiments were performed at different combinations. The central composite experimental plan along with the predicted and observed response for each individual experiment is summarized in Table 6. It shows the production of cellulase (U/ml) corresponding to combined effect of all three components in the specified ranges. The production of cellulase may be best predicted by the following model:

5 $$ \begin{gathered} {\text{Cellulase }} = \, \left( { 2 7. 3 2} \right) \, + \, \left( { 1. 2 8 {\text{A}}} \right) \, + \, \left( {0. 8 6 {\text{B}}} \right) \, - \, \left( { 2. 2 7 {\text{C}}} \right) \, - \, \left( {0. 8 5 {\text{AB}}} \right) + \, \left( {0. 9 8 {\text{AC}}} \right) \, \hfill \\ - \, \left( {0.00 6 {\text{BC}}} \right) \, - \, \left( {0. 4 1 {\text{A2}}} \right) \, - \, \left( { 1. 9 4 {\text{B2}}} \right) \, - \, \left( { 1. 3 3 {\text{C2}}} \right) \hfill \\ \end{gathered} $$

**Table 6:** Full experimental central composite design with coded and actual level of variables and the response function

	A: CMC g %		B: MgSO_4_		Ph		Cellulase activity U/mL	
Run	Actual	Coded	Actual	Coded	Actual	Coded	Actual	Predicted
1	0.5	−1	0.05	−1	7	−1	23.6	23.88629
2	1.5	+1	0.05	−1	7	−1	24.55	26.20009
3	0.5	−1	0.5	+1	7	−1	25.05	27.348
4	1.5	+1	0.5	+1	7	−1	24.85	26.23679
5	0.5	−1	0.05	−1	10	+1	16.85	17.38949
6	1.5	+1	0.05	−1	10	+1	24	23.62829
7	0.5	−1	0.5	+1	10	+1	20.55	20.8262
8	1.5	+1	0.5	+1	10	+1	22	23.63999
9	0.159104	−α	0.275	0	8.5	0	25.1	24.00702
10	1.840896	+α	0.275	0	8.5	0	29.95	28.3188
11	1	0	−0.1034	–α	8.5	0	20.7	20.37752
12	1	0	0.653403	+α	8.5	0	25.7	23.2983
13	1	0	0.275	0	5.977311	−α	29.8	27.3863
14	1	0	0.275	0	11.02269	+α	20.05	19.73953
15	1	0	0.275	0	8.5	0	24.3	27.31957
16	1	0	0.275	0	8.5	0	29	27.31957
17	1	0	0.275	0	8.5	0	28.7	27.31957
18	1	0	0.275	0	8.5	0	24.75	27.31957
19	1	0	0.275	0	8.5	0	26.9	27.31957
20	1	0	0.275	0	8.5	0	29.8	27.31957

where, Y is cellulase production (U/mL); A is CMC concentration (g%); B is MgSO4 (g%); C is pH concentration.

**Fig. 2:**
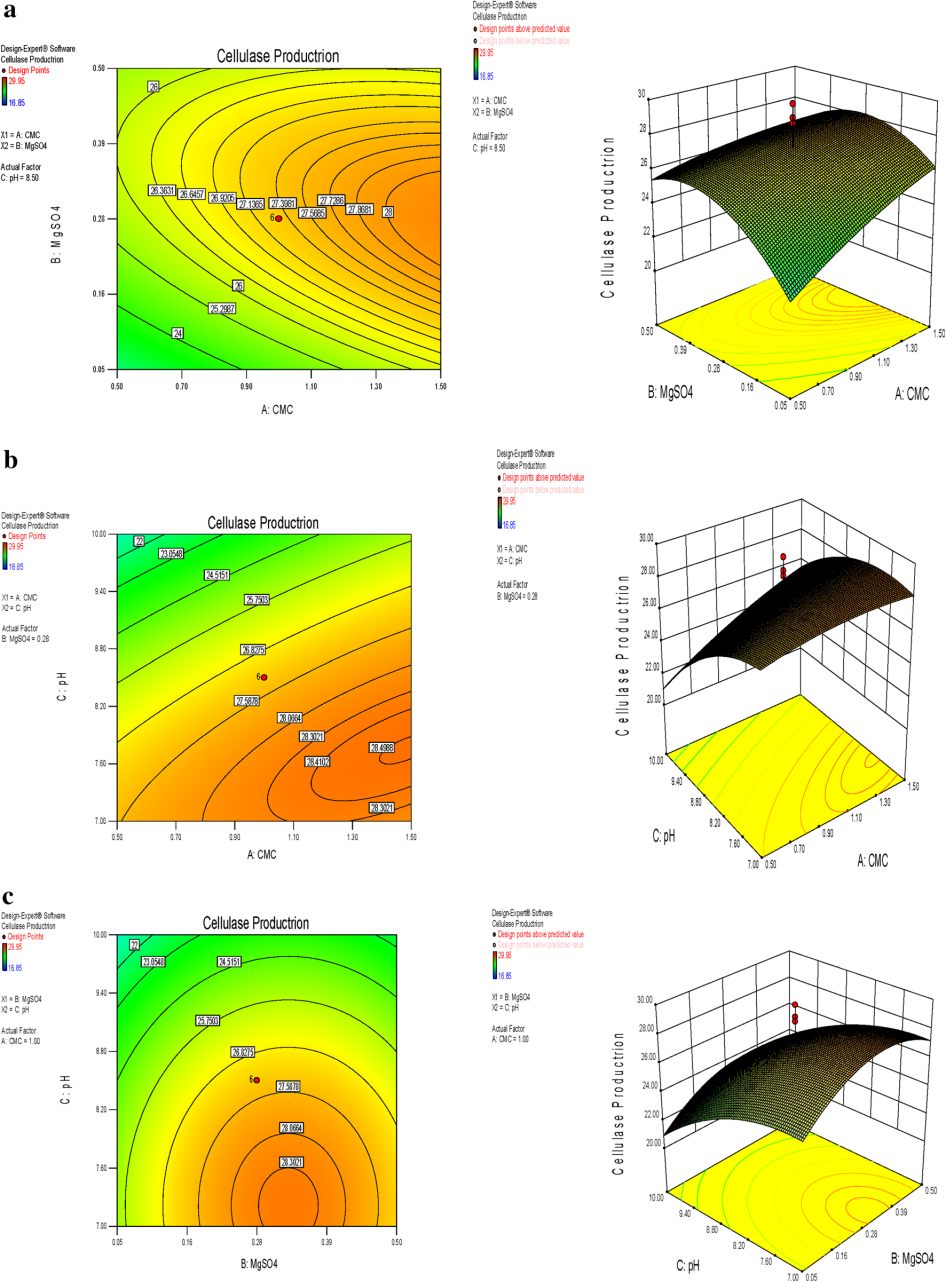
Response surface graph showing interaction effects between concentration of **a** CMC and MgSO4. **b** pH and CMC. **c** MgSO4 and pH

The statistical significance of the second-order polynomial equation was evaluated by F-test ANOVA which revealed that this regression is statistically highly significant for cellulase production. The model F-value of 3.79 implies that the model is significant. There is only a 2.47 % chance that a large ‘model F-value’ could occur due to noise. Values of ‘prob > F’ less than 0.050 indicate that the model terms are significant (Table 7). The ‘lack-of-fit F-value’ of 1.08 implies the lack of fit is not significant relative to the pure error. There is 46.72 % chance that a large ‘lack-of-fit F-value’ could occur due to noise. Non-significant lack of fit is good for the model to fit. The R2 value (multiple correlation coefficient) closer to 1 denotes better correlation between observed and predicted values. The coefficient of variation (CV) indicates the degree of precision with which the experiments are compared. The lower reliability of the experiment is usually indicated by high value of CV. In the present case a low CV (9.51) denotes that the experiments performed are reliable. Adequate precision measures the signal to noise ratio. A ratio greater than 4 is desirable. In our case, the ratio is of 6.553, which indicates an adequate signal. This model can be used to navigate the design space. The effect of interaction of variables on enzyme (cellulase production) yield was studied against any two independent variables while keeping the other independent variables at their constant level. These response surface plots or contour plots can be used to predict the optimal values for different test variables. Therefore, three response surfaces were obtained by considering all the possible combinations. Three-dimensional response plot shown in Fig. 2a describes the behavior of cellulase production, main effect, interaction effect, and squared effect (nonlinear) of MgSO_4_ and pH at different concentrations. The shape of the response surface curves showed a moderate interaction between these tested variables. As observed in the contour plot, the middle level of MgSO_4_ and middle level of CMC resulted in higher enzyme production. It has been reported that CMC shows inducing effect on cellulase production. Biosynthesis of cellulases in *Trichoderma reesei* was very high in medium with carboxymethylcellulose as carbon source [12].The three-dimensional curve and contour plot of the calculated response surface from the interaction between CMC and pH while keeping fixed concentration of MgSO_4_ are shown in Fig. 2b. Both components at their lower level did not result in the higher enzyme yield while CMC at the higher level and pH at their lower level showed the maximum enzyme activity. Li et al. [13] also reported the importance of the substrate concentration for xylanse production by *Aspergillus awamori*. These facts might be accounted for by the report that the enzymes involved in substrate degradation were generally inducible and were formed only when the corresponding substrate was present in the nutrient solution. The interaction plot of MgSO_4_ and pH is shown in Fig. 2c, where the shape of the response surface indicates the interaction of MgSO_4_ and pH with the fixed coded value of CMC. An increasing MgSO_4_ with simultaneous decrease in pH let to increase in cellulase. The enzyme yield was found to increase with higher level of MgSO_4_ with lower level of pH. Cui and Zhao [14] reported the xylanase production could achieve a higher activity than 44 U/mL when the concentration of MgSO_4_ was at a higher level between −0.3 and 0.1 (coded value). It is possible that Mg^2+^ has a positive effect on the stabilization of the ribosome and cellular membranes and Ca^2+^ plays a protector role in the medium, which relatively enhances the activity of xylanase. Some researchers have suggested the importance of Mg^2+^ as a trace element for xylanase production by *Aspergillus fischeri* Fxn 1 and by *Streptomyces olivaceoviridis* E-86, and other researchers found that Mg^2+^ combined with Fe^2+^ played an important part in xylanase production by *Streptomyces* sp. QG-11-3, and that Zn^2+^, Mg^2+^ combining with Fe^2+^ could stimulate the yield of xylanase by *Streptomyces thermodiasticus*. RSM has been employed for the production of lactic acid from wheat bran by using *Lactobacillus amylophilus* GV6 [15, 16]. It has also been applied for the production of various enzymes, such as cyclodextrin glucanotransferase (CGTase) [17], chitinase [18], α-amylase [19], pectinase [20], Lipase [21] and vitamin riboflavin [22]. RSM can also be useful in optimizing the enzyme reaction conditions. A second order polynomial equation was found to be useful for the development of efficient bioprocess for cellulase production. Second-order polynomial models were used to correlate the abovementioned factors to soluble periplasmic IFN-α2b formation and percentage of soluble IFN-α2b translocated to the periplasmic space of *E. coli* and the models were found to be significant and subsequently validated [23]. Statistical optimization of fermentation conditions were reported to enhanced the cellulase production to 2995.20 ± 200.00 IU/mL, which was 9.91-fold higher than the activity under unoptimized basal medium (302.2 IU/mL) [24]. The biosynthesis of cellulases in *Trichoderma reesei* was very high in medium with carboxymethylcellulose as carbon source [12]. Similarly maximum amount of chitinase (3.47 U/mL) was produced by *Basidiobolus ranarum* with 1.5 % colloidal chitin, 0.125 % lactose, 0.025 % malt extract and 0.075 % peptone [25]. Li et al. [26] reported maximum cellulase activity (0.26 U/mL) of a *Bacillus* sp. When the culture was grown in LB medium supplemented with 1 % CMC. It has been reported recently that *Bacillus* sp. (DUSELR13) and *Brevibacillus* sp. (DUSELG12) isolated from gold mine produced maximum CMCase activity 0.12 and 0.02 U/mL, respectively, under unoptimized conditions [27]. However Cellulase enzyme from *B. subtilis* (AS3) had an optimum pH of 9.2 so it was called alkaline cellulase [1]. Considering this property of our enzyme (which is alkaline in nature) can be used for various applications. The optimum formula of cellulase production from marine isolate *Streptomyces ruber* was KH_2_PO_4_ 1.5 (g/L), MgSO_4_ 0.1(g/L), MnSO_4_ 0.05(g/L), NH_4_NO_3_ 0.5(g/L), NaCl 1.5(g/L) with inoculum size of 0.5 mL [28]. RSM based on CCD was also used to optimize the media for cellulase production using four medium constituents, OPEFB fibers (5–15 g/L), yeast extract (3–9 g/L), CaCl2 (1–5 mM), MgSO4 (3–7 mM) and to optimize the level of two environmental condition agitation speed (200–300 rpm) and temperature (28–32 °C) from *Aspergillus terreus* [29]. The production of endoglucanase was studied by *Aspergillus terreus* by applying the Plackett-Burman design for optimization of process parameters and this study agreed with our results in that, both KH_2_SO_4_ and MgSO_4_ positively affected CMCase production. High levels of KH_2_SO_4_ and low levels of MgSO_4_, maximized the enzyme production [30].

**Table 7:** Analysis of variance of quadratic model for cellulase production using MBAA3

Source	Sum of	df	Mean	F	*P* value	
	squares		square	value	Prob > F	
Model	189.9858	9	21.10953213	3.79448	0.0247	Significant
A-CMC	22.4418	1	22.44179898	4.033958	0.0724	
B-MgSO_4_	10.29776	1	10.29776171	1.851043	0.2035	
C-pH	70.58331	1	70.58331317	12.68749	0.0052	
AB	5.865313	1	5.8653125	1.054302	0.3287	
AC	7.702812	1	7.7028125	1.384596	0.2666	
BC	0.000312	1	0.0003125	5.62E−05	0.9942	
A^2	2.410015	1	2.410015225	0.433205	0.5253	
B^2	54.12976	1	54.1297625	9.729932	0.0109	
C^2	25.42233	1	25.42232541	4.569713	0.0583	
Residual	55.63221	10	5.56322108			
Lack of fit	28.89013	5	5.778025493	1.080324	0.4672	Not significant
Pure error	26.74208	5	5.348416667			
Core total	245.618	19

### Validation of the Model

Validation was carried out under conditions predicted by the model. The optimal concentrations estimated for each variable were 1.84 g CMC, 0.275 g MgSO_4_ and pH 8.5. The predicted cellulase production obtained from the model using the above optimum concentration of medium components was 29.95 U/mL. To validate the prediction of the model, additional experiments in triplicate were performed with the optimized medium. These experiments yielded the maximum cellulase activity of 30.62 U/mL. Agreements between the predicted and experimental results verified the validity of the model and the existence of the optimal points.

The data obtained after optimization has resulted in 29.95 U/mL enzyme production. After optimization cellulase activity increased by 6.81 fold. The statistical design of experiment offer efficient methodology to identify the significant variables and to optimize the factors with minimum number of experiments for cellulase production by microorganism. These significant factors identified by Plackett-Burman design were considered for the next stage in the medium optimization by using response surface optimization technique. The conversion of cellulosic biomass by microorganisms is a potential sustainable approach to develop novel bioprocesses and products.

### Isolation and Quantification of Genomic DNA

**Fig. 3:**
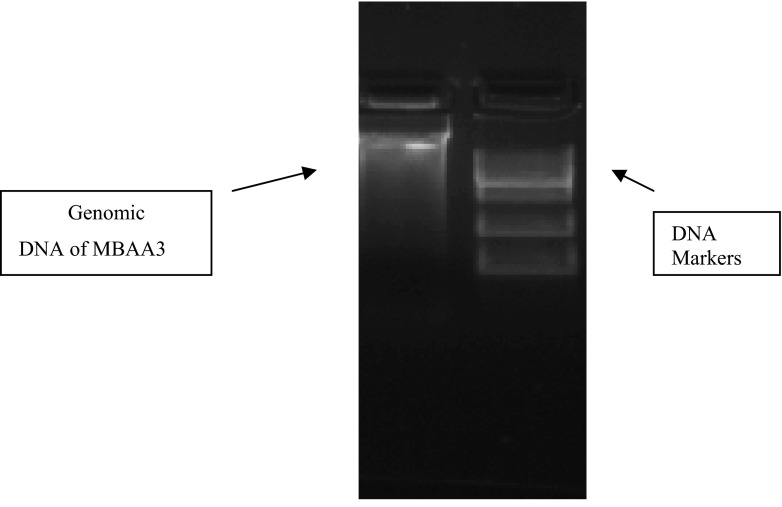
Genomic DNA of MBAA3

For the cellulase gene detection the genomic DNA of MBAA3 was isolated [31] and concentration was 0.498 mg/mL was confirmed by gel electrophoresis shown in Fig. 3.

### PCR Amplification of Cellulase Genes

**Fig. 4:**
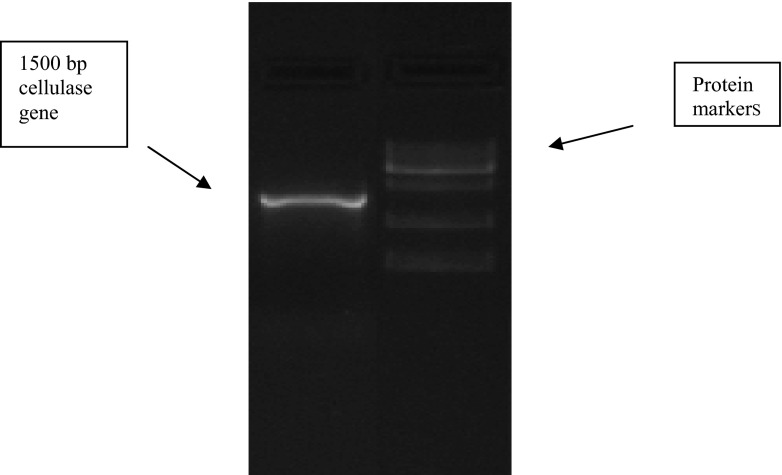
Amplification of cellulases gene from MBAA3. *Lane I*: amplified cellulase gene using CelF & CelR primers *Lane II*: DNA marker at annealing temperature of 58 °C

Amplification of cellulase gene among several sets of primers tried during the study, the best amplification was observed by the primer set CelF This CelF: 5′-ATGAAACGGTCAATCTC-3′ and CelR 5′-CTAATTTGGTTCTGTTCCC-3′ set of primer was tried following the sequence of cellulase gene from *B. amyloliquefaciens* PSM 3.1 [32]. Among rest of sets of primers, sufficient amplification of cellulase gene could not be achieved as some set of primers showed non specific amplification of gene while a few others amplified DNA fragments of very minute base pair size (~ 300). The amplification of 1500 bp gene of cellulase by primer set CelF & CelR is shown in Fig. 4.

Cellulase gene detection screening showed that *Cel* genes was present in the MBAA3 and PCR products were sequenced and analyzed using the National center of biotechnological information nBlast database. The PCR product showed very high homology to the nearest sequence of the respective gene. Moreover, a protein BLAST search indicated that this gene show similarity to protein sequence of cellulase. The nucleotide sequence of cellulase gene was submitted in the gene bank under the accession number KF929416.

The PCR products obtained using *B. amyloliquefaciens* PSM 3.1 chromosomal DNA as template were about 1500 bp and other non-specific bands were obtained [32]. Amplification of cellulase gene was reported in which sequence specific primers for cellulase genes were used and significant amplification was achieved from the cDNA and the corresponding bands were found in the gel [33]. The results of this study may further be exploited for characterization of a recombinant cellulase enzyme after its expression in a suitable host to produce novel cellulases with high efficiency. The biological aspects of processing of cellulosic biomass become the crux of future research involving cellulases and cellulolytic microorganisms. With modern biotechnology tools, especially in the area of microbial genetics, novel enzymes and new enzyme applications will become available for the various industries.

## Experimental Section

### Strain Isolation and Identification

Bacterial culture isolated from farm soil samples collected from Ahmedabad, Gujarat, India on nutrient agar (Himedia, Mumbai, India) was screened for cellulase production on CMC agar plate. The screening was done by streaking the isolated colonies on the plates containing 1 % CMC. After 24 h incubation the plates were flooded with 0.1 % congo red solution and left undisturbed for 15 min. To visualize clear zones formed by cellulase positive strains the plates were destained using 1 M NaCl solution [34]. Positive and better zone producing strain was chosen and continued for further studies. Culture was maintained at 4 °C N.agar (peptone 5 g/L, NaCl 5 g/L, beef extract 1.5 g/L, yeast extract 1.5 g/L, agar 15 g/L, pH 7.4) slants containing 1 % CMC. Genomic DNA of the isolate was extracted and the 16S rDNA gene was amplified using universal primers. The purified PCR product was sequenced and the phylogenic relationship of the isolate was determine by comparing the sequence data with the existing sequences available through the gene bank database of the National Center for Biotechnology Information (NCBI, Bethesda, MD, USA) and the sequence was submitted in the gene bank.

### Plackett- Burman Experimental Design

In the first step of the optimization process Plackett-Burman experimental design was used to identify the significance of the ingredients of the media for the optimum production of cellulase enzyme. Plackett-Burman statistical experimental design is very useful and widely employed in the screening of major constituents of the media [35]. This design gave an output of 12 experimental runs with eight independent variables (Table 1). All the experiments were performed in triplicate and the average of cellulase activity was used as the response (dependant variable) [36]. The main effect of each variable was calculated as the difference between the average of measurements made at the high value (+) and at the low value (−) (Table 2). This model does not describe the interaction among the factors and it is used to evaluate and select the important factors that influence the response. The effects of individual parameters on cellulase production were calculated by the following equation:

1 $$ {\text{E}}\left( {\text{Xi}} \right) = 2\left( {{\text{M}} + {\text{M}} - } \right)/{\text{N}} $$

where, E is the effect of parameter under study; M+ and M− are responses (cellulase) of trials at which the parameter was at its higher and lower levels, respectively; N is the total number of trials.

Experimental error was estimated by calculating the variance among the dummy variables as

2 $$ {\text{Veff}} = \varSigma \left( {\text{Ed}} \right) 2/{\text{n}} $$

where, Veff is the variance of the effect of level; Ed is the effect of level for the dummy variables; and n is the number of dummy variables used in the experiment. The standard error (SE, Es) of concentration effect was the square root of variance of an effect, and the significance level (p-value) of each concentration effect was determined using the student’s t-test:

3 $$ {\text{t}}\left( {\text{Xi}} \right) = {\text{E}}\left( {\text{Xi}} \right)/{\text{Es}} $$

where, E(Xi) is the effect of variable Xi.

### Response Surface Methodology (RSM)

The next step in formulation of the medium was to determine the optimum levels of significant variables for enhancing cellulase production [37]. For this purpose, RSM using CCD was employed. CCD consisting of 20 experimental runs, with 5 axial points (α ± 2) and 3 replications at the central point (0), was employed to optimize the concentration of significant factors. The design space consisted of three variables, which are CMC, MgSO_4_ and pH as they showed significant positive effect on cellulase production during analysis of Plackett−Burman statistical design. RSM is useful for small number of variables (up to 5) but is impractical for large number of variables, due to high number of experimental runs required. According to the design, the total number of treatment combinations is 2^k^+ 2k + η_o_, where k is the number of independent variables and η_o_ is the number of repetition of experiments at the central point. Each factor in the design was studied at five different levels (−α, − 1, 0, +1, +α) as shown in Table 3. The minimum and maximum ranges of variables were determined on the basis of our previous experiments. The full experimental plan with respect to their values in actual and coded form is listed in Table 6. Enzymatic activity was measured in triplicate in 20 different experimental runs. The cellulase production was analyzed by using a second order polynomial equation, and the data were fitted into the equation by multiple regression procedure. The model equation for analysis is given as:

4 $$ Y = \beta 0 + \beta iXi + \beta iiXi2 + \beta ijXiXj $$

where, β0, βi, βii, and βij represent the constant process effect in total, the linear, quadratic effect of Xi, and the interaction effect between Xi and Xj, respectively for the production of cellulase. Later, an experiment was run using the optimum values for variables given by response optimization to confirm the predicted value and cellulase production was confirmed. Flask with 100 mL of autoclaved production medium inoculated with 1 mL of culture was incubated in rotary shaker at 200 rpm at 37 °C for 48 h. The DNS assay was carried out as follows to determine cellulase activity. 0.5 mL of culture filtrate was mixed with 0.5 mL 1 % CMC in phosphate buffer pH 7 in a test tube and incubated at 40 °C for 30 min. The reaction was terminated by adding 2 mL of DNS reagent. The tube was then incubated in boiling waterbath for 15 min The OD was taken at 540 nm against blank. One unit of the cellulase activity refers to the amount of enzyme that released 1 μg of glucose under assay condition [38].

### Software and Data Analysis

The results of the experimental design were analyzed and interpreted using Design-Expert version 8.0 (Stat-Ease Inc., Minneapolis, MN, USA) statistical software.

### Isolation of DNA for Cellulase Gene Amplification

For cellulase gene detection the isolate was grown aerobically overnight at 37 °C in LB broth. Bacterial chromosomal DNA was purified using Genomic DNA Purification Kit (Xcelgen).

### Quantization and Quality Assessment of DNA

The DNA stock samples was quantified using nanodrop spectrophotometer at 260 and 280 nm using the convention that one absorbance unit at 260 nm wavelength equals 50 µg DNA per ml. Purity of DNA was judged on the basis of optical density ratio at 260:280 nm [32]. The DNA having ratio between 1.8 and 2.0 was considered to be of good purity. Concentration of DNA was estimated using the formula. 

Concentration of DNA (mg/mL) = OD 260 × 50 × Dilution factor. 

Quality and purity of DNA were checked by agarose gel electrophoresis. Agarose 0.8 % (w/v) in 0.5X TAE (pH 8.0) buffer was used for submarine gel electrophoresis. Ethidium bromide (1 %) was added @ 10 µl/100 mL. The wells were charged with 5 µL of DNA preparations mixed with 1 µL gel loading dye. Electrophoresis was carried out at 80 V for 30 min at room temperature. DNA was visualized under UV using UV transilluminator. The DNA was used further for amplification of cellulase gene.

### Polymerase Chain Reaction

For the confirmation of cellulase gene the gene fragment was amplified by PCR from genomic DNA using gene specific set of primers: celF (5′-ATGAAACGGTCAATCTC-3′) and celR (5′**-**CTAATTTGGTTCTGTTCCC-3′) [32]. PCR was carried out in a final reaction volume of 25 µl in 200 µL capacity thin wall PCR tube in Eppendorf Thermal Cycler. Reaction mixture for PCR tubes containing the mixture were tapped gently and spin briefly at 10000 rpm. The PCR tubes with all the components were transferred to thermal cycler. The PCR protocol designed for 30 cycles for the primers used is given in Table 4.

### Visualization and Purification of PCR Product

To confirm the targeted PCR amplification, 5 µL of PCR product from each tube was mixed with 1 µL of 6 ×  gel loading dye and electrophoresed on 1.2 % agarose gel containing ethidium bromide (1 per cent solution @10 µL/100 mL) at constant 5 V/cm for 30 min in 0.5 × TAE buffer. The amplified product was visualized as a single compact band of expected size under UV light and documented by gel documentation system (Biorad). Length of the product is approx.1500 bp Amplified PCR product was purified using Qiagen Mini elute Gel extraction kit according to the manufactures protocol.

### Sequencing of Purified Gene Segment

The concentration of the purified DNA was determined and was subjected to automated DNA sequencing on ABI 3730xl Genetic Analyzer (Applied Biosystems, USA). Sequencing was carried out using BigDye^®^ Terminator v3.1 Cycle sequencing kit following manufacturer instructions. Cycle sequencing was performed following the instructions supplied along with BigDye^®^ Terminator v3.1 Cycle Sequencing Kit. The reaction was carried out in a final reaction volume of 20 µL using 200 µL capacity thin wall PCR tube. The cycling protocol was designed for 25 cycles Denaturation at 96 °C for 10 s, Annealing at 58 °C for 5 s and Extension at 60 °C for 4 min with the thermal ramp rate of 1 °C per second. After cycling, the extension products were purified and mixed well in 10 µL of Hi-Di formamide. The contents were mixed on shaker for 30 min at 300×g. Eluted PCR products were placed in a sample plate and covered with the septa. Sample plate was heated at 95 °C for 5 min, snap chilled and loaded into autosampler of the instrument.

Electrophoresis and data analysis was carried out on the ABI 3730xl Genetic Analyzer using appropriate Module, Basecaller, Dyeset/Primer and Matrix files.
